# The *CCND1* c.870G risk allele is enriched in individuals of African ancestry with plasma cell dyscrasias

**DOI:** 10.1038/s41408-020-0294-5

**Published:** 2020-03-16

**Authors:** Linda B. Baughn, Zhuo Li, Kathryn Pearce, Celine M. Vachon, Mei-Yin Polley, Jonathan Keats, Eran Elhaik, Michael Baird, Terry Therneau, James R. Cerhan, P. Leif Bergsagel, Angela Dispenzieri, S. Vincent Rajkumar, Yan W. Asmann, Shaji Kumar

**Affiliations:** 1 Division of Laboratory Genetics, Department of Laboratory Medicine and Pathology, Mayo Clinic, Rochester, MN USA; 2 Division of Biomedical Statistics and Informatics, Department of Health Sciences Research, Mayo Clinic, Jacksonville, FL USA; 3 Division of Biomedical Statistics and Informatics, Department of Health Sciences Research, Mayo Clinic, Rochester, MN USA; 4 Integrated Cancer Genomics, Translational Genomics Research Institute (TGen), Phoenix, AZ USA; 5 Department of Biology, Lund University, Lund, Sweden; 6 DNA Diagnostics Center, Fairfield, OH USA; 7 Division of Hematology, Department of Internal Medicine, Mayo Clinic, Scottsdale, AZ USA; 8 Division of Hematology, Department of Internal Medicine, Mayo Clinic, Rochester, MN USA

**Keywords:** Cancer genetics, Risk factors, Myeloma

Dear Editor,

Plasma cell (PC) dyscrasias, including multiple myeloma (MM), represent a spectrum of monoclonal gammopathies resulting from a clonal expansion of an abnormal plasma cell clone^1^. MM is generally preceded by precursor conditions known as monoclonal gammopathy of undetermined significance (MGUS) and smoldering MM (SMM) and is the most common hematologic malignancy in African Americans (AAs)^2,3^. It has been established that AAs, including Ghanaian men, display a 2–3-fold higher prevalence of MGUS with a reported similar risk of MGUS to MM progression compared to European Americans (EAs)^4–6^. An increased MM incidence has also been observed in individuals with a family history of MM, an effect that was greater for individuals who self-identify as black^7^, suggesting that constitutional, MM susceptibility risk allele/s may explain the increased prevalence of MGUS among individuals of African ancestry. Interestingly, AAs also appear to have either a similar or in some cases superior survival outcome compared to EAs depending on patient age, specific treatment modalities and access to care including clinical trial enrollment^3,8–10^. This disparity in outcome may be explained by genetic differences that predispose to specific acquired, cytogenetically defined subtypes, which can influence disease prognosis and response to treatment. These cytogenetic subtypes can be broadly classified into either hyperdiploid (characterized by gains of odd-numbered chromosomes) or translocations involving the immunoglobulin heavy chain (*IgH*) gene on chromosome 14. In the context of MM, hyperdiploidy and translocations t(11;14)(q13;q32) and t(6;14)(p21;q32) are typically associated with a favorable prognosis, while translocations t(4;14)(p16;q32), t(14;16)(q32;q23), or t(14;20)(q32;q12) translocations are generally associated with an unfavorable prognosis^1^.

Many previous studies evaluating the mechanism of racial disparities in MGUS/MM have assessed race using self-reported demographic data rather than utilizing ancestry informative single-nucleotide polymorphisms (SNPs) as a method to determine the proportion of racial admixture. Using SNP data, we have recently identified a higher prevalence of *IgH* translocations t(11;14), t(14;16) and t(14;20) in individuals with ≥80% African ancestry^11^. Enrichment of t(11;14) was confirmed in a more recent study^12^. As an extension to our previous work^11^, we investigated which, if any, of the germline risk alleles previously associated with MM or MGUS risk, survival or risk of development of t(11;14)^13^ (Supplemental Table 1) are enriched in individuals of African ancestry in our cohort of patients with plasma cell dyscrasias.

Genotype and ancestry information were obtained from the Precision Medicine Research Array (PMRA) data of 898 samples from patients with an abnormal plasma cell proliferative disorder fluorescence in situ hybridization (FISH) result and a concurrent conventional G-banded chromosome study (Supplemental materials and methods). Eight-hundred eighty-one of these samples were previously described^11^. As expected, the patient demographics of the full 898 sample cohort were similar to prior results^11^, including the increased risk of development of either a t(11;14), t(14;16) or t(14;20) in individuals with high African ancestry (≥80%, very African cohort) compared to individuals with low African ancestry (<0.1%, <30% Asian ancestry, European cohort) (Supplemental Tables 2 and 3).

Of the 18 previously reported SNPs examined within our cohort (Supplementary Table 1), 11 risk alleles were associated with European ancestry (<0.1% African and <30% Asian ancestries), involving variants at 2p23.3, 2q12.3, 3q26.2, 5q15, 6p21.33, 6q21, 10p12.1, 16q23.1, and 17p11.2 (Supplemental Table 4). Seven risk alleles were found associated with African ancestry (≥80% African ancestry) involving variants at 3p22.1, 3q26.2, 7p15.3, 8q24.21, 11q13.3, 16p13.11, and 17p11.2 (Supplemental Table 5). Of these seven variants, rs9344 at 11q13.3 encoding the *CCND1* c.870G > A polymorphism was of particular interest due to the enrichment of the G-risk allele among persons of African ancestry (Table 1A, *p*-value < 0.0001) and its association with increased risk of t(11;14)^13^.

**Table 1:** rs9344 SNP in association with African ancestry in A and with t(11;14) in B.

**(A**)											
	Gene	Allele		SNP genotype associations with race					*p*-value		
	Chr.	Risk	Other	GT	European *N* = 238	Other *N* = 537	Very African *N* = 123	Total *N* = 898	Overall	European vs. very African	Other vs. very African
				Missing	4	5	3	12			
rs9344	CCND1	G	A	AA	39 (16.7%)	81 (15.2%)	5 (4.2%)	125 (14.1%)	<0.0001	<0.0001	<0.0001
	11q13.3			AG	112 (47.9%)	266 (50.0%)	35 (29.2%)	413 (46.6%)			
				GG	83 (35.5%)	185 (34.8%)	80 (66.7%)	348 (39.3%)			
		Risk allele frequency			0.59	0.60	0.81	0.63			

(B)				
	Non-t(11;14) (*N* = 637)	t(11;14) (*N* = 261)	Total (*N* = 898)	*p*-value
rs9344				<0.0001
Missing	6	6	12	
AA	109 (87.2%)	16 (12.8%)	125 (14.1%)	
AG	308 (74.6%)	105 (25.4%)	413 (46.6%)	
GG	214 (61.5%)	134 (38.5%)	348 (39.3%)	
Risk allele frequency	0.58	0.73	0.63	

A Chi-squared test was used to evaluate the differences across these groups.

European (<0.1% African ancestry and <30% Asian ancestry), other (not European or African), very African (≥ 80% African ancestry). Chi-squared test was used to determine the overall comparison between the three ancestral groups and also between ancestral groups 1 vs. 2, and 2 vs. 3 using pairwise comparison. Pairwise comparison difference is considered statistically significant when *p* < 0.025 based on Bonferroni method for multiple comparison.

Of the seven risk alleles associated with African ancestry, only rs9344 was shown to be associated with t(11;14) in our full patient cohort with a G-risk allele frequency of 0.58 in non-t(11;14) controls compared to 0.73 in t(11;14) cases (Table 1B, *p*-value < 0.0001). The six additional risk alleles associated with African ancestry were not found to be associated with t(11;14) (Supplemental Table 6). The G allele correlated with t(11;14) in both European (*p*-value = 0.0048) and African (*p*-value = 0.0522) cohorts (Supplemental Table 7). A multivariate model including age, gender, race, and rs9344 identified rs9344 as significantly associated with t(11;14) after adjusting for age, gender, and race group (Table 2) (*p*-value < 0.001 for GG and *p*-value = 0.005 for AG genotypes).

**Table 2:** Multivariate model predicting t(11;14).

Variable	Class	OR 95 CI	*p*-value
Age		1.01 (0.99, 1.02)	0.338
Gender	Male	1.57 (1.15, 2.14)	0.004
rs9344	AG genotype	2.27 (1.28, 4.04)	0.005
	GG genotype	4.02 (2.26, 7.17)	<0.001
Race	Very African	1.17 (0.69, 1.97)	0.564
	Other	1.04 (0.72, 1.49)	0.845

Variables included age, gender, race, and SNP rs9344

other (not European or African), very African (≥80% African ancestry). A logistic regression model was used to evaluate the differences.

To test if these results are replicable, we employed the Multiple Myeloma Research Foundation (MMRF) CoMMpass cohort, which includes individuals with newly diagnosed MM. Although the t(11;14) was not enriched in self-reported black individuals from this cohort (Supplemental Table 8) (17.5% Black vs. 20.4% White, *p*-value = 0.47), there was a significant association between the SNP rs9344 and self-reported black race (Supplemental Tables 8 and 9) (*p*-value < 0.0001) and with t(11;14) when the t(11;14) was analyzed as both total number of supporting read pairs (*p*-value = 0.0004) and presence or absense of t(11;14) identified by long insert whole-genome sequencing (Supplemental Table 9) (*p*-value = 0.0001).

Here, we evaluated the prevalence of 18 previously published germline MGUS/MM risk alleles in our cohort of 898 patients stratified by genetically inferred ancestry rather than self-reported race data. Using this stratification approach, we confirm the association of the *CCND1* c.870G risk allele (SNP rs9344) with cytogenetic subtype t(11;14) in both European and African populations. The frequency of the G-risk allele in our European cohort was similar to the reported risk allele frequency in the UK and German cohorts^13^. Our study is the first to demonstrate that the G-risk allele is associated with African ancestry. This overall increased frequency of the G-risk allele in individuals with the highest African ancestry (0.81) vs. lowest African ancestry (0.59) (*p* < 0.0001) suggests that this risk allele may contribute to the increased prevalence of t(11;14) in AAs. Two other SNPs rs649392 and rs1352075 within *CCND1* that are in linkage disequilibrium with rs9344 were also significantly correlated with t(11;14) and African ancestry (data not shown). No significant correlation was found between *CCND1* SNPs rs9344, rs649392, or rs1352075 in association with t(14;16) or (14;20) cytogenetic subtypes (data not shown).

Although we validate the association between rs9344 and self-reported black race and with t(11;14) in the CoMMpass study, we did not observe increased t(11;14) with self-reported black race. One possibility for this finding may be the presence of a smaller number of patients that self-reported as black with very high African ancestry. As reported by Manojlovic et al.^9^ there were <20 patients with ≥80% African ancestry in the CoMMpass IA9 dataset. Given that the *CCND1* rs9344 polymorphism is a common allele and does not contribute to t(11;14) risk in a fully penetrant manner, it is probable that additional variants present in individuals with ≥80% African ancestry also contribute t(11;14) risk.

The *CCND1* gene encodes the cyclin D1 protein, a member of the cyclin D family, which regulate cyclin-dependent kinases (Cdks). Cyclin D in concert with Cdks phosphorylate the retinoblastoma protein promoting cell cycle progression^14^. MM tumors typically deregulate one of three cyclin D proteins^15^ and t(11;14) translocation is one mechanism promoting cyclin D1 deregulation^16^. The c.870G > A polymorphism is at chr11:69,462,910 (GRCh37/hg19) of the last nucleotide of exon 4 of *CCND1* (NM_053056). Although both 870G and 870A encode the proline amino acid, the G allele has been reported to cause a novel splice donor site resulting in a longer cyclin D1a transcript^14^. The A allele has been reported to hinder splicing of exon 4 resulting in intron 4 read-through creating a truncated D1b transcript producing a protein shorter than D1a and lacking the D1a carboxy-terminus^14^. The lack of the carboxy-terminus of the D1b isoform results in nuclear retention^17^. Most studies have associated the A allele with cancer risk and poor outcome, with only a few studies correlating the G allele to increased cancer risk^14^. Of interest, the G allele has been associated with increased risk of cervical, head and neck and colorectal cancers, malignancies AAs have also been reported with increased incidence compared to EAs^14^.

To our knowledge, our study includes the largest group of individuals of African ancestry with an abnormal plasma cell clone along with uniformly collected FISH, genotyping, and ancestry data. Utilization of the immunoglobulin kappa and lambda light chain counterstain during FISH analysis provide specificity allowing for the scoring of only light chain expressing plasma cells clones. Inclusion of only cytogenetically abnormal clones with recurrent MM abnormalities provides addition specificity at the risk of excluding clones with normal or non-recurring cytogenetic abnormalities that our plasma cell FISH panels do not detect. Utilization of this population of patients with abnormal FISH studies, we have identified the association of the G-risk allele of *CCND1* c.870G > A polymorphism (rs9344) with African ancestry and with t(11;14), suggesting that it may play a role in the development of t(11;14) plasma cell disorders. Future studies are needed to further understand the mechanism behind this association and also evaluate whether there exists differences in outcome and/or response to therapy between AAs and EAs with t(11;14) MM.

## Supplementary information

Supplemental materials and methods
