# Supplementary material for: The CCND1 c.870G risk allele is enriched in individuals of African ancestry with plasma cell dyscrasias
Source: Blood Cancer J. 2020 Mar 16;10(3):39. doi: 10.1038/s41408-020-0294-5 (PMC7075993; doi:10.1038/s41408-020-0294-5)
Supplement: Supplementary file 1 — Supplemental materials and methods [file 41408_2020_294_MOESM1_ESM.docx]

**The *CCND1* 870G risk allele is enriched in individuals of African ancestry with plasma cell dyscrasias**

Linda B. Baughn, Ph.D.^1^, Zhuo Li, M.S.^2^ , Kathryn Pearce, M.S.^1^, Celine M. Vachon, Ph.D. ^3^, Mei-Yin Polley, P.D. ^3^, Jonathan Keats, Ph.D ^4^, Eran Elhaik, Ph.D.^5^, Michael Baird, Ph.D.^6^, Terry Therneau, Ph.D.^3^, James R. Cerhan, M.D., Ph.D. ^3^, P. Leif Bergsagel, M.D. ^7^, Angela Dispenzieri, M.D.^8^, S. Vincent Rajkumar, M.D. ^8^, Yan W. Asmann, Ph.D.^2^ , Shaji Kumar, M.D.^8^*

^1^Division of Laboratory Genetics, Department of Laboratory Medicine and Pathology, Mayo Clinic, Rochester, MN, ^2^Division of Biomedical Statistics and Informatics, Department of Health Sciences Research, Mayo Clinic, Jacksonville, FL, ^3^Division of Biomedical Statistics and Informatics, Department of Health Sciences Research, Mayo Clinic, Rochester, MN, ^4^Integrated Cancer Genomics, Translational Genomics Research Institute (TGen), Phoenix, AZ. ^5^Department of Biology, Lund University, Lund, Sweden, ^6^DNA Diagnostics Center, Fairfield, OH, ^7^Division of Hematology, Department of Internal Medicine, Mayo Clinic, Scottsdale, AZ, ^8^Division of Hematology, Department of Internal Medicine, Mayo Clinic, Rochester, MN.

* Corresponding author.

Division of Hematology, Department of Internal Medicine, Mayo Clinic, 200 First Street SW, Rochester, MN 55905 (Kumar.Shaji@Mayo.edu)

**Supplementary Materials and Methods**

**Study cohort**

A total of 898 samples were obtained from the Mayo Clinic Genomics Laboratory after obtaining an Institutional Review Board approval. Of these samples, 881 were described previously (1). All 898 samples were obtained from a retrospective cohort of specimens from patients who had an abnormal plasma cell proliferative disorder fluorescence in situ hybridization (FISH) result and a concurrent conventional G-banded chromosome evaluation as part of routine clinical testing. As in (1), we also recorded the abnormal plasma cell FISH result, patient age at the time of clinical cytogenetic testing, gender, and self-reported race (if available). Any duplicate patient samples and samples with only an abnormal monosomy 15 fluorescence in situ hybridization (FISH) result were removed. Monosomy 15 identified by FISH is a reported polymorphism and does not represent an MM-specific genomic abnormality (2).

**FISH and conventional chromosome analysis**

Plasma cell proliferative disorder FISH of immunoglobulin (cIg)-stained positive plasma cells were performed as part of routine clinical testing as described (1). Plasma cell specificity was achieved using immunoglobulin staining with antibodies targeting cytoplasmic immunoglobulin kappa and lambda proteins. A conventional G-banded chromosome evaluation was performed as part of routine clinical testing (1).

**DNA Extraction and PMRA Genotyping**

DNA was isolated from fixed cell pellets from residual chromosome studies (1). Genotyping was performed on a 96-well Precision Medicine Research Array (PMRA) (ThermoFisher Scientific, <https://www.thermofisher.com/order/catalog/product/902981>) with ~730 000 autosomal single nucleotide polymorphisms (SNPs), following the manufacturer’s recommended protocol. One negative and two positive controls (Coriell samples) were included on each run. Data were analyzed by the Axiom Analysis Software Suite (ThermoFisher Scientific) to determine genotypes with a required call rate threshold of at least 99%. For quality control, arrays with a dish QC (dQC) value greater than or equal to 0.82 and a QC call rate of 97% or greater are considered to be passed. More details regarding QC can be found in the Axiom Genotyping Solution Data Analysis Guide (<https://tools.thermofisher.com/content/sfs/manuals/axiom_genotyping_solution_analysis_guide.pdf>)

## **Biogeographical inference**

Biogeographical analyses were carried out using the commercial Geographic Population Structure Origins (GPSO) tool provided by the DNA Diagnostics Center (3-5), which calculates the ancestry of an individual and outputs admixture proportions corresponding to those ancestries (4).

**SNP imputation**

Eighteen established SNPs for either MM or MGUS risk, t(11;14) MM risk or MM survival were curated from the literature with emphasis on genomic regions reported in Rand et al (Supplemental Table 1) (6-16) and tested for associations with African ancestry. Nine SNPs were not included in the PMRA. These SNPs were imputed to 1,000 Genomes (17) phase 3 dataset (all populations) using IMPUTE2 (18) to obtain alternative SNPs in LD with the MM SNPs (*r^2^* > 0.6, 500-kb flanking).

**Statistical analysis and calculation of odds ratios**

Continuous variables were summarized using mean (standard deviation) and median (range) while categorical variables were reported as frequency (percentage). To examine the association between ancestry percentage with SNPs and MM abnormalities, individuals were divided into three ancestral groups: 1. European (<0.1% African ancestry and <30% Asian ancestry); 2. Very African (> 80% African ancestry); and 3. Other. Chi-squared test was used to determine the overall comparison between the 3 ancestral groups and also between ancestral groups 1 vs 2, and 2 vs 3 using pairwise comparison.  In addition, multivariate logistic regression was performed to adjust for age and gender. All tests were two-sided with alpha level set at 0.05 for overall statistical significance. Pairwise comparison was considered statistically significant when p<0.025 based on Bonferroni method for multiple comparisons.

**Multiple Myeloma Research Foundation (MMRF) CoMMpass validation cohort**

A total of 689 CoMMpass study patients with self-reported black or white race were included as a validation cohort. The germline exome sequencing data in Sequence Read Archive (SRA) format were downloaded from dbGaP (<https://www.ncbi.nlm.nih.gov/gap>, phs000748) and converted to FASTQ files. The reads were aligned to Human Reference Genome build 37 (GRCh37/hg19) using BWA-MEM (19). Single nucleotide variants and short insertions and deletions were identified using GATK (20) version 3.4 following the Broad Institute’s germline short variant discovery best practice guidelines (<https://software.broadinstitute.org/gatk/best-practices/workflow?id=11145>). The germline and MM tumor long insert whole genome sequencing data from these 689 patients were also downloaded and processed by a Mayo in-house analytic pipeline for identification of somatic large structural variants including translocations, insertions, inversions, and large INDELs (Asmann *et al.,* manuscript in preparation). Translocations such as t(11;14) were represented either qualitatively (with or without t(11;14)) or quantitatively (number of discordant read pairs supporting t(11;14)).

**Supplementary Table 1: Characteristics of the germline SNPs evaluated in this study**

|  | | | **SNP** | **Imputed from index SNP** | | | **Known association** | **Chr** | | **Position** | | **Gene** | **Risk allele** | | | **Other** | |
| --- | --- | --- | --- | --- | --- | --- | --- | --- | --- | --- | --- | --- | --- | --- | --- | --- | --- |
| 1 | | | rs7577599 |  | | |  | 2p23.3 | | 25,613,146 | | DTNB | T | | | C | |
|  |  |  | in LD with (*r^2^*) | rs6761076 (0.791) | | | MM/MGUS | 2p23.3 | | 25,607,758 | | DTNB | T | | | C | |
| 2 | | | rs6746082 |  | | | MM/MGUS | 2p23.3 | | 25,659,244 | | DTNB | A | | | C | |
| 3 | | | rs1524295 |  | | |  | 2q12.3 | | 107,622,046 | | Intergenic | T | | | C | |
|  |  |  | in LD with (*r^2^*) | rs13416655 (0.947) | | | MM/MGUS | 2q12.3 | | 107,621,925 | | Intergenic | C | | | T | |
| 4 | | | rs55650463 |  | | |  | 2q12.3 | | 107,634,954 | | Intergenic | G | | | T | |
|  |  |  | in LD with (*r^2^*) | rs12614346 (0.623) | | | MM/MGUS | 2q12.3 | | 107,642,482 | | Intergenic | A | | | G | |
| 5 | | | rs1052501 |  | | | MM/MGUS | 3p22.1 | | 41,925,398 | | ULK4 | C | | | T | |
| 6 | | | rs10936599 |  | | | MM/MGUS | 3q26.2 | | 169,492,101 | | MYNN | C | | | T | |
| 7 | | | rs6793295 |  | | |  | 3q26.2 | | 169,518,455 | | LRRC34 | T | | | C | |
|  |  |  | in LD with (*r^2^*) | rs9811216 (0.758) | | | MM/MGUS | 3q26.2 | | 169,487,501 | | ACTRT3 | T | | | C | |
| 8 | | | rs2161318 |  | | |  | 5q15 | | 95,288,576 | | ELL2 | T | | | C | |
|  |  |  | in LD with (*r^2^*) | rs6877329 (0.686) | | | MM/MGUS | 5q15 | | 95,261,538 | | ELL2 | C | | | G | |
| 9 | | | rs2285803 |  | | | MM/MGUS | 6p21.33 | | 31,107,258 | | PSORS1C1 | T | | | C | |
| 10 | | | rs9372120 |  | | | MM/MGUS | 6q21 | | 106,667,535 | | ATG5 | G | | | T | |
| 11 | | | rs57104699 |  | | |  | 7p15.3 | | 21,928,079 | | DNAH11 | C | | | A | |
|  |  |  | in LD with (*r^2^*) | rs12540021 (0.712) | | | MM/MGUS | 7p15.3 | | 21,945,563 | | CDCA7L | G | | | A | |
| 12 | | | rs1948915 |  | | | MM/MGUS | 8q24.21 | | 128,222,421 | | CCAT1 | C | | | T | |
| 13 | | | rs2790455 |  | | |  | 10p12.1 | | 28,847,077 | | WAC | A | | | G | |
|  |  |  | in LD with (*r^2^*) | rs2790457 (0.976) | | | MM/MGUS | 10p12.1 | | 28,856,819 | | WAC | G | | | A | |
| 14 | | | rs9344/  rs603965 |  | | | t(11;14) MM | 11q13.3 | | 69,462,910 | | CCND1 | G | | | A | |
| 15 | | | rs10656021 |  | | |  | 16p13.11 | | 15,967,362-15,967,364 | | FOPNL | CTAdup | | | CTA | |
|  |  |  | in LD with (*r^2^*) | rs72773978 (0.652) | | | MM Survival | 16p13.11 | | 15,974,642 | | FOPNL | T | | | A | |
| 16 | | | rs7193541 |  | | | MM/MGUS | 16q23.1 | | 74,664,743 | | RFWD3 | T | | | C | |
| 17 | | | rs4985726 |  | | |  | 17p11.2 | | 16,863,638 | | TNFRSF13B | G | | | C | |
|  |  |  | in LD with (*r^2^*) | rs34562254 (0.626) | | | MM/MGUS | 17p11.2 | | 16,842,991 | | TNFRSF13B | A | | | G | |
| 18  Previously reported index SNPs or alternative SNPs when index SNP was not available on the PMRA. Chr: chromosome band location of SNP and position in GRCh37/hg19. | | | rs4273077 |  | | | MM/MGUS | 17p11.2 | | 16,849,139 | | TNFRSF13B | G | | | A | |
| **Supplementary Table 2. Patient demographics by race** | | | | | | | | | | | | | |  |  |  |  |
|  | | | **European (N=238)** | **Other (N=537)** | | | **Very African (N=123)** | | **Total (N=898)** | | | **Overall** | | | **European vs very African** | | **Other vs very African** |
| **Age** | | |  |  | | |  | |  | | | 0.0078 | | | 0.0202 | | 0.7658 |
| Mean (SD) | | | 64.9 (10.4) | 62.4 (10.9) | | | 62.2 (11.0) | | 63.1 (10.8) | | |  | | |  | |  |
| Median | | | 65.0 | 64.0 | | | 62.0 | | 64.0 | | |  | | |  | |  |
| Q1, Q3 | | | 58.0, 72.0 | 55.0, 70.0 | | | 56.0, 69.0 | | 56.0, 70.0 | | |  | | |  | |  |
| Range | | | (32.0-90.0) | (26.0-89.0) | | | (37.0-84.0) | | (26.0-90.0) | | |  | | |  | |  |
|  | | |  |  | | |  | |  | | |  | | |  | |  |
| **Gender** | | |  |  | | |  | |  | | | 0.0258 | | | 0.0088 | | 0.0182 |
| Female | | | 99 (41.6%) | 238 (44.3%) | | | 69 (56.1%) | | 406 (45.2%) | | |  | | |  | |  |
| Male | | | 139 (58.4%) | 299 (55.7%) | | | 54 (43.9%) | | 492 (54.8%) | | |  | | |  | |  |

European (<0.1% African ancestry and <30% Asian ancestry), Other (not European or African), Very African (> 80% African ancestry). Chi-squared test was used to determine the overall comparison between the 3 ancestral groups and also between ancestral groups 1 vs 2, and 2 vs 3 using pairwise comparison. Pairwise comparison difference is considered statistically significant when p<0.025 based on Bonferroni method for multiple comparison.

| **Supplementary Table 3. Patient primary cytogenetic subtype by race** | | | | |  |  |  |  |
| --- | --- | --- | --- | --- | --- | --- | --- | --- |
|  | **European (N=238)** | **Other (N=537)** | **Very African (N=123)** | **Total (N=898)** | | **Overall** | **European vs very African** | **Other vs very African** |
| **t(11:14) or t(14:16) or t(14, 20)** |  |  |  |  | | 0.0067 | 0.0051 | 0.0026 |
| No | 158 (66.4%) | 353 (65.7%) | 63 (51.2%) | 574 (63.9%) | |  |  |  |
| Yes | 80 (33.6%) | 184 (34.3%) | 60 (48.8%) | 324 (36.1%) | |  |  |  |
|  |  |  |  |  | |  |  |  |
| **t(11;14)** |  |  |  |  | | 0.0893 | 0.049 | 0.0382 |
| No | 173 (72.7%) | 387 (72.1%) | 77 (62.6%) | 637 (70.9%) | |  |  |  |
| Yes | 65 (27.3%) | 150 (27.9%) | 46 (37.4%) | 261 (29.1%) | |  |  |  |
|  |  |  |  |  | |  |  |  |
| **t(14,16) or t(14,20)** |  |  |  |  | | 0.1246 | 0.0924 | 0.0517 |
| No | 223 (93.7%) | 503 (93.7%) | 109 (88.6%) | 835 (93.0%) | |  |  |  |
| Yes | 15 (6.3%) | 34 (6.3%) | 14 (11.4%) | 63 (7.0%) | |  |  |  |
|  |  |  |  |  | |  |  |  |
| **Hyperdiploid only without IGH separation** |  |  |  |  | | 0.1796 | 0.0664 | 0.1218 |
| No | 141 (59.2%) | 331 (61.6%) | 85 (69.1%) | 557 (62.0%) | |  |  |  |
| Yes | 97 (40.8%) | 206 (38.4%) | 38 (30.9%) | 341 (38.0%) | |  |  |  |

European (<0.1% African ancestry and <30% Asian ancestry), Other (not European or African), Very African (> 80% African ancestry). Chi-squared test was used to determine the overall comparison between the 3 ancestral groups and also between ancestral groups 1 vs 2, and 2 vs 3 using pairwise comparison. Pairwise comparison difference is considered statistically significant when p<0.025 based on Bonferroni method for multiple comparison.

**Supplemental table 4: Germline susceptibility SNPs in association with European ancestry**

| **SNPs** | **Gene** | **Allele** | | **SNP Genotype Associations with Race** | | | |  | **p-value** | | |
| --- | --- | --- | --- | --- | --- | --- | --- | --- | --- | --- | --- |
|  | **Chr.** | **Risk** | **Other** | **GT** | **European** | **Other** | **Very African** | **Total** | Overall | European vs very African | Other vs very African |
| rs7577599 | DTNB | T | C | CC | 7 (3.0%) | 33 (6.2%) | 16 (13.4%) | 56 (6.3%) | <0.0001 | <0.0001 | <0.0001 |
|  | 2p23.3 |  |  | CT | 57 (24.3%) | 156 (29.5%) | 53 (44.5%) | 266 (30.1%) |  |  |  |
|  |  |  |  | TT | 171 (72.8%) | 340 (64.3%) | 50 (42.0%) | 561 (63.5%) |  |  |  |
| rs6746082 | DTNB | A | C | CC | 9 (3.8%) | 32 (6.0%) | 21 (17.6%) | 62 (7.0%) | <0.0001 | <0.0001 | <0.0001 |
|  | 2p23.3 |  |  | CA | 71 (30.2%) | 191 (36.0%) | 57 (47.9%) | 319 (36.0%) |  |  |  |
|  |  |  |  | AA | 155 (66.0%) | 308 (58.0%) | 41 (34.5%) | 504 (56.9%) |  |  |  |
| rs1524295 | Intergenic | T | C | CC | 58 (24.7%) | 142 (26.6%) | 44 (36.7%) | 244 (27.5%) | 0.1287 | 0.038 | 0.0648 |
|  | 2q12.3 |  |  | CT | 124 (52.8%) | 280 (52.5%) | 58 (48.3%) | 462 (52.0%) |  |  |  |
|  |  |  |  | TT | 53 (22.6%) | 111 (20.8%) | 18 (15.0%) | 182 (20.5%) |  |  |  |
| rs55650463 | Intergenic | G | T | TT | 119 (50.6%) | 309 (58.0%) | 104 (86.7%) | 532 (59.9%) | <0.0001 | <0.0001 | <0.0001 |
|  | 2q12.3 |  |  | TG | 96 (40.9%) | 185 (34.7%) | 16 (13.3%) | 297 (33.4%) |  |  |  |
|  |  |  |  | GG | 20 (8.5%) | 39 (7.3%) | 0 (0.0%) | 59 (6.6%) |  |  |  |
| rs6793295 | LRRC34 | T | C | CC | 17 (7.2%) | 54 (10.1%) | 17 (14.2%) | 88 (9.9%) | 0.0008 | 0.0001 | 0.0082 |
|  | 3q26.2 |  |  | CT | 81 (34.5%) | 214 (40.2%) | 62 (51.7%) | 357 (40.2%) |  |  |  |
|  |  |  |  | TT | 137 (58.3%) | 265 (49.7%) | 41 (34.2%) | 443 (49.9%) |  |  |  |
| rs2161318 | ELL2 | T | C | CC | 10 (4.3%) | 67 (12.6%) | 37 (30.8%) | 114 (12.8%) | <0.0001 | <0.0001 | <0.0001 |
|  | 5q15 |  |  | CT | 76 (32.3%) | 181 (34.0%) | 59 (49.2%) | 316 (35.6%) |  |  |  |
|  |  |  |  | TT | 149 (63.4%) | 285 (53.5%) | 24 (20.0%) | 458 (51.6%) |  |  |  |
| rs2285803 | PSORS1C1 | T | C | CC | 86 (36.8%) | 276 (51.9%) | 62 (51.7%) | 424 (47.9%) | 0.0029 | 0.026 | 0.8605 |
|  | 6p21.33 |  |  | CT | 122 (52.1%) | 215 (40.4%) | 47 (39.2%) | 384 (43.3%) |  |  |  |
|  |  |  |  | TT | 26 (11.1%) | 41 (7.7%) | 11 (9.2%) | 78 (8.8%) |  |  |  |
| rs9372120 | ATG5 | G | T | TT | 148 (63.0%) | 362 (67.9%) | 106 (88.3%) | 616 (69.4%) | <0.0001 | <0.0001 | <0.0001 |
|  | 6q21 |  |  | TG | 72 (30.6%) | 149 (28.0%) | 14 (11.7%) | 235 (26.5%) |  |  |  |
|  |  |  |  | GG | 15 (6.4%) | 22 (4.1%) | 0 (0.0%) | 37 (4.2%) |  |  |  |
| rs2790455 | WAC | A | G | GG | 10 (4.3%) | 54 (10.2%) | 30 (25.2%) | 94 (10.6%) | <0.0001 | <0.0001 | <0.0001 |
|  | 10p12.1 |  |  | GA | 92 (39.1%) | 234 (44.1%) | 55 (46.2%) | 381 (43.1%) |  |  |  |
|  |  |  |  | AA | 133 (56.6%) | 243 (45.8%) | 34 (28.6%) | 410 (46.3%) |  |  |  |
| rs7193541 | RFWD3 | T | C | CC | 31 (13.2%) | 98 (18.4%) | 32 (26.7%) | 161 (18.1%) | 0.0197 | 0.004 | 0.1161 |
|  | 16q23.1 |  |  | CT | 111 (47.2%) | 259 (48.6%) | 54 (45.0%) | 424 (47.7%) |  |  |  |
|  |  |  |  | TT | 93 (39.6%) | 176 (33.0%) | 34 (28.3%) | 303 (34.1%) |  |  |  |
| rs4985726 | TNFRSF13B | G | C | CC | 188 (80.7%) | 394 (73.9%) | 111 (92.5%) | 693 (78.2%) | 0.0002 | 0.0115 | 0.0001 |
|  | 17p11.2 |  |  | CG | 42 (18.0%) | 122 (22.9%) | 9 (7.5%) | 173 (19.5%) |  |  |  |
|  |  |  |  | GG | 3 (1.3%) | 17 (3.2%) | 0 (0.0%) | 20 (2.3%) |  |  |  |

European (<0.1% African ancestry and <30% Asian ancestry), Other (not European or African), Very African (> 80% African ancestry). Chr: chromosome band location of SNP. Risk: risk allele. GT: genotype. Chi-squared test was used to determine the overall comparison between the 3 ancestral groups and also between ancestral groups 1 vs 2, and 2 vs 3 using pairwise comparison. Pairwise comparison difference is considered statistically significant when p<0.025 based on Bonferroni method for multiple comparison.

**Supplemental table 5: Germline susceptibility SNPs in association with African ancestry**

| **SNPs** | **Gene** | **Allele** | | **SNP Genotype Associations with Race** | | | | | **p-value** | | |
| --- | --- | --- | --- | --- | --- | --- | --- | --- | --- | --- | --- |
|  | **Chr.** | **Risk** | **Other** | **GT** | **European** | **Other** | **Very African** | **Total** | Overall | European vs very African | Other vs very African |
| **rs9344*** | CCND1 | G | A | AA | 39 (16.7%) | 81 (15.2%) | 5 (4.2%) | 125 (14.1%) | <0.0001 | <0.0001 | <0.0001 |
|  | 11q13.3 |  |  | AG | 112 (47.9%) | 266 (50.0%) | 35 (29.2%) | 413 (46.6%) |  |  |  |
|  |  |  |  | GG | 83 (35.5%) | 185 (34.8%) | 80 (66.7%) | 348 (39.3%) |  |  |  |
| **rs1052501** | ULK4 | C | T | TT | 152 (65.0%) | 248 (46.6%) | 9 (7.6%) | 409 (46.2%) | <0.0001 | <0.0001 | <0.0001 |
|  | 3p22.1 |  |  | TC | 72 (30.8%) | 214 (40.2%) | 52 (43.7%) | 338 (38.2%) |  |  |  |
|  |  |  |  | CC | 10 (4.3%) | 70 (13.2%) | 58 (48.7%) | 138 (15.6%) |  |  |  |
| **rs10936599** | MYNN | C | T | TT | 16 (6.8%) | 31 (5.8%) | 1 (0.8%) | 48 (5.4%) | <0.0001 | <0.0001 | <0.0001 |
|  | 3q26.2 |  |  | TC | 74 (31.5%) | 159 (29.8%) | 10 (8.3%) | 243 (27.4%) |  |  |  |
|  |  |  |  | CC | 145 (61.7%) | 343 (64.4%) | 109 (90.8%) | 597 (67.2%) |  |  |  |
| **rs57104699** | DNAH11 | C | A | AA | 20 (8.5%) | 34 (6.4%) | 0 (0.0%) | 54 (6.1%) | <0.0001 | <0.0001 | <0.0001 |
|  | 7p15.3 |  |  | AC | 96 (40.9%) | 167 (31.4%) | 11 (9.2%) | 274 (30.9%) |  |  |  |
|  |  |  |  | CC | 119 (50.6%) | 331 (62.2%) | 109 (90.8%) | 559 (63.0%) |  |  |  |
| **rs1948915** | CCAT1 | C | T | TT | 99 (42.1%) | 175 (33.0%) | 13 (10.8%) | 287 (32.4%) | <0.0001 | <0.0001 | <0.0001 |
|  | 8q24.21 |  |  | TC | 111 (47.2%) | 238 (44.8%) | 43 (35.8%) | 392 (44.2%) |  |  |  |
|  |  |  |  | CC | 25 (10.6%) | 118 (22.2%) | 64 (53.3%) | 207 (23.4%) |  |  |  |
| **rs10656021** | FOPNL | CTAdup | CTA | CTA | 207 (88.5%) | 487 (91.7%) | 97 (80.8%) | 791 (89.4%) | 0.0019 | 0.0511 | 0.0004 |
|  | 16p13.11 |  |  | CTAdup | 27 (11.5%) | 44 (8.3%) | 23 (19.2%) | 94 (10.6%) |  |  |  |
| **rs4273077** | TNFRSF13B | G | A | AA | 191 (81.3%) | 379 (71.1%) | 95 (79.2%) | 665 (74.9%) | 0.0165 | 0.6503 | 0.1032 |
|  | 17p11.2 |  |  | AG | 40 (17.0%) | 134 (25.1%) | 24 (20.0%) | 198 (22.3%) |  |  |  |
|  |  |  |  | GG | 4 (1.7%) | 20 (3.8%) | 1 (0.8%) | 25 (2.8%) |  |  |  |

European (<0.1% African ancestry and <30% Asian ancestry), Other (not European or African), Very African (> 80% African ancestry). Chr: chromosome band location of SNP. Risk: risk allele. GT: genotype. SNP rs9344 indicated by a *. Chi-squared test was used to determine the overall comparison between the 3 ancestral groups and also between ancestral groups 1 vs 2, and 2 vs 3 using pairwise comparison. Pairwise comparison difference is considered statistically significant when p<0.025 based on Bonferroni method for multiple comparison.

**Supplemental table 6: Correlation between other SNPs enriched in individuals of African ancestry and t(11;14) in full patient cohort**

|  | **Non-t(11;14) (N=637)** | **t(11;14) (N=261)** | **Total (N=898)** | **p-value** |
| --- | --- | --- | --- | --- |
| **rs1052501** |  |  |  | 0.2660 |
| Missing | 9 | 4 | 13 |  |
| TT | 299 (73.1%) | 110 (26.9%) | 409 (46.2%) |  |
| TC | 238 (70.4%) | 100 (29.6%) | 338 (38.2%) |  |
| CC | 91 (65.9%) | 47 (34.1%) | 138 (15.6%) |  |
| **rs10936599** |  |  |  | 0.8110 |
| Missing | 6 | 4 | 10 |  |
| TT | 36 (75.0%) | 12 (25.0%) | 48 (5.4%) |  |
| TC | 171 (70.4%) | 72 (29.6%) | 243 (27.4%) |  |
| CC | 424 (71.0%) | 173 (29.0%) | 597 (67.2%) |  |
| **rs57104699** |  |  |  | 0.5405 |
| Missing | 6 | 5 | 11 |  |
| AA | 35 (64.8%) | 19 (35.2%) | 54 (6.1%) |  |
| AC | 194 (70.8%) | 80 (29.2%) | 274 (30.9%) |  |
| CC | 402 (71.9%) | 157 (28.1%) | 559 (63.0%) |  |
| **rs1948915** |  |  |  | 0.6658 |
| Missing | 8 | 4 | 12 |  |
| TT | 209 (72.8%) | 78 (27.2%) | 287 (32.4%) |  |
| TC | 273 (69.6%) | 119 (30.4%) | 392 (44.2%) |  |
| CC | 147 (71.0%) | 60 (29.0%) | 207 (23.4%) |  |
| **rs10656021** |  |  |  | 0.0603 |
| Missing | 8 | 5 | 13 |  |
| CTA | 570 (72.1%) | 221 (27.9%) | 791 (89.4%) |  |
| CTAdup | 59 (62.8%) | 35 (37.2%) | 94 (10.6%) |  |
| **rs4273077** |  |  |  | 0.8537 |
| Missing | 6 | 4 | 10 |  |
| AA | 471 (70.8%) | 194 (29.2%) | 665 (74.9%) |  |
| AG | 141 (71.2%) | 57 (28.8%) | 198 (22.3%) |  |
| GG | 19 (76.0%) | 6 (24.0%) | 25 (2.8%) |  |

A Chi-squared test was used to evaluate the differences across these groups.

**Supplemental table 7: Correlation between rs9344, t(11;14) and ancestry**

|  |  | **Non-t(11;14)** | **t(11;14)** | **p-value** |
| --- | --- | --- | --- | --- |
| **European** | **rs9344** |  |  | 0.0048 |
|  | AA | 35 (89.7%) | 4 (10.3%) |  |
|  | AG | 84 (75.0%) | 28 (25.0%) |  |
|  | GG | 52 (62.7%) | 31 (37.3%) |  |
|  | Risk allele frequency | **0.55** | **0.71** |  |
| **Very African** | **rs9344** |  |  | 0.0522 |
|  | AA | 4 (80.0%) | 1 (20.0%) |  |
|  | AG | 27 (77.1%) | 8 (22.9%) |  |
|  | GG | 44 (55.0%) | 36 (45.0%) |  |
|  | Risk allele frequency | **0.77** | **0.89** |  |
| **Other** | **rs9344** |  |  | 0.0005 |
|  | AA | 70 (86.4%) | 11 (13.6%) |  |
|  | AG | 197 (74.1%) | 69 (25.9%) |  |
|  | GG | 118 (63.8%) | 67 (36.2%) |  |
|  | Risk allele frequency | **0.56** | **0.69** |  |

European (<0.1% African ancestry and <30% Asian ancestry), Other (not European or African), Very African (> 80% African ancestry). A Chi-squared test was used to evaluate the differences across these groups.

**Supplemental table 8: Patient demographics, rs9344 and t(11;14) status in MMRF CoMMpass cohort based on self-report race**

|  | **White (N=569)** | **Black (N=120)** | **Total (N=689)** | **p-value** |
| --- | --- | --- | --- | --- |
| **Age** |  |  |  | 0.1968 |
| N | 569 | 120 | 689 |  |
| Mean (SD) | 64.6 (10.6) | 63.0 (12.6) | 64.3 (11.0) |  |
| Median | 65.0 | 63.0 | 65.0 |  |
| Q1, Q3 | 57.0, 71.0 | 54.0, 72.0 | 57.0, 71.0 |  |
| Range | (27.0-93.0) | (32.0-90.0) | (27.0-93.0) |  |
|  |  |  |  |  |
| **Gender** |  |  |  | 0.8129 |
| Female | 221 (38.8%) | 48 (40.0%) | 269 (39.0%) |  |
| Male | 348 (61.2%) | 72 (60.0%) | 420 (61.0%) |  |
|  |  |  |  |  |
| **rs9344** |  |  |  | <0.0001 |
| GG | 194 (34.1%) | 77 (64.2%) | 271 (39.3%) |  |
| AG | 273 (48.0%) | 39 (32.5%) | 312 (45.3%) |  |
| AA | 102 (17.9%) | 4 (3.3%) | 106 (15.4%) |  |
|  |  |  |  |  |
| **Sum of chr11:14 reads** |  |  |  | 0.3887 |
| N | 569 | 120 | 689 |  |
| Mean (SD) | 6.2 (15.4) | 5.4 (20.8) | 6.0 (16.5) |  |
| Median | 0.0 | 0.0 | 0.0 |  |
| Q1, Q3 | 0.0, 0.0 | 0.0, 0.0 | 0.0, 0.0 |  |
| Range | (0.0-131.0) | (0.0-203.0) | (0.0-203.0) |  |
|  |  |  |  |  |
| **t(11;14)** |  |  |  | 0.4715 |
| No | 453 (79.6%) | 99 (82.5%) | 552 (80.1%) |  |
| Yes | 116 (20.4%) | 21 (17.5%) | 137 (19.9%) |  |

A Chi-squared test was used to evaluate the differences across these groups.

**Supplemental table 9: Patient demographics, self-report race and t(11;14) status in MMRF CoMMpass cohort based on rs9344 genotype**

| **rs9344** | | | | | |
| --- | --- | --- | --- | --- | --- |
|  | **GG (N=271)** | **AG (N=312)** | **AA (N=106)** | **Total (N=689)** | **p-value** |
| **Age** |  |  |  |  | 0.4415 |
| N | 271 | 312 | 106 | 689 |  |
| Mean (SD) | 64.1 (11.3) | 64.3 (10.8) | 65.2 (11.1) | 64.3 (11.0) |  |
| Median | 64.0 | 65.0 | 66.0 | 65.0 |  |
| Q1, Q3 | 56.0, 71.0 | 57.5, 71.0 | 58.0, 72.0 | 57.0, 71.0 |  |
| Range | (27.0-93.0) | (31.0-90.0) | (36.0-91.0) | (27.0-93.0) |  |
|  |  |  |  |  |  |
| **Gender** |  |  |  |  | 0.2932 |
| Female | 109 (40.2%) | 113 (36.2%) | 47 (44.3%) | 269 (39.0%) |  |
| Male | 162 (59.8%) | 199 (63.8%) | 59 (55.7%) | 420 (61.0%) |  |
|  |  |  |  |  |  |
| **Self-reported Black** |  |  |  |  | <0.0001 |
| No | 194 (71.6%) | 273 (87.5%) | 102 (96.2%) | 569 (82.6%) |  |
| Yes | 77 (28.4%) | 39 (12.5%) | 4 (3.8%) | 120 (17.4%) |  |
|  |  |  |  |  |  |
| **Sum of chr11:14 reads** |  |  |  |  | 0.0004 |
| N | 271 | 312 | 106 | 689 |  |
| Mean (SD) | 8.3 (20.3) | 4.4 (12.0) | 5.2 (16.4) | 6.0 (16.5) |  |
| Median | 0.0 | 0.0 | 0.0 | 0.0 |  |
| Q1, Q3 | 0.0, 8.0 | 0.0, 0.0 | 0.0, 0.0 | 0.0, 0.0 |  |
| Range | (0.0-203.0) | (0.0-73.0) | (0.0-108.0) | (0.0-203.0) |  |
|  |  |  |  |  |  |
| **t(11:14)** |  |  |  |  | 0.0001 |
| No | 196 (72.3%) | 263 (84.3%) | 93 (87.7%) | 552 (80.1%) |  |
| Yes | 75 (27.7%) | 49 (15.7%) | 13 (12.3%) | 137 (19.9%) |  |

A Chi-squared test was used to evaluate the differences across these groups.

**References**

1. Baughn LB, Pearce K, Larson D, Polley MY, Elhaik E, Baird M, et al. Differences in genomic abnormalities among African individuals with monoclonal gammopathies using calculated ancestry. Blood Cancer J. 2018;8(10):96.
2. Smadbeck J, Peterson JF, Pearce KE, Pitel BA, Figueroa AL, Timm M, et al. Mate pair sequencing outperforms fluorescence in situ hybridization in the genomic characterization of multiple myeloma. Blood cancer J. 2019:9(12):103.
3. Elhaik E, Yusuf L, Anderson AIJ, Pirooznia M, Arnellos D, Vilshansky G, et al. The Diversity of REcent and Ancient huMan (DREAM): A New Microarray for Genetic Anthropology and Genealogy, Forensics, and Personalized Medicine. Genome Biol Evol. 2017;9(12):3225-37.
4. Das R, Wexler P, Pirooznia M, Elhaik E. Localizing Ashkenazic Jews to Primeval Villages in the Ancient Iranian Lands of Ashkenaz. Genome Biol Evol. 2016;8(4):1132-49.
5. Elhaik E, Tatarinova T, Chebotarev D, Piras IS, Maria Calo C, De Montis A, et al. Geographic population structure analysis of worldwide human populations infers their biogeographical origins. Nat Commun. 2014;5:3513.
6. Broderick P, Chubb D, Johnson DC, Weinhold N, Forsti A, Lloyd A, et al. Common variation at 3p22.1 and 7p15.3 influences multiple myeloma risk. Nat Genet. 2011;44(1):58-61.
7. Weinhold N, Johnson DC, Chubb D, Chen B, Forsti A, Hosking FJ, et al. The CCND1 c.870G>A polymorphism is a risk factor for t(11;14)(q13;q32) multiple myeloma. Nat Genet. 2013;45(5):522-5.
8. Chubb D, Weinhold N, Broderick P, Chen B, Johnson DC, Forsti A, et al. Common variation at 3q26.2, 6p21.33, 17p11.2 and 22q13.1 influences multiple myeloma risk. Nat Genet. 2013;45(10):1221-5.
9. Greenberg AJ, Lee AM, Serie DJ, McDonnell SK, Cerhan JR, Liebow M, et al. Single-nucleotide polymorphism rs1052501 associated with monoclonal gammopathy of undetermined significance and multiple myeloma. Leukemia. 2013;27(2):515-6.
10. Weinhold N, Johnson DC, Rawstron AC, Forsti A, Doughty C, Vijayakrishnan J, et al. Inherited genetic susceptibility to monoclonal gammopathy of unknown significance. Blood. 2014;123(16):2513-7.
11. Ziv E, Dean E, Hu D, Martino A, Serie D, Curtin K, et al. Genome-wide association study identifies variants at 16p13 associated with survival in multiple myeloma patients. Nat Commun. 2015;6:7539.
12. Swaminathan B, Thorleifsson G, Joud M, Ali M, Johnsson E, Ajore R, et al. Variants in ELL2 influencing immunoglobulin levels associate with multiple myeloma. Nat Commun. 2015;6:7213.
13. Mitchell JS, Li N, Weinhold N, Forsti A, Ali M, van Duin M, et al. Genome-wide association study identifies multiple susceptibility loci for multiple myeloma. Nat Commun. 2016;7:12050.
14. Rand KA, Song C, Dean E, Serie DJ, Curtin K, Sheng X, et al. A Meta-analysis of Multiple Myeloma Risk Regions in African and European Ancestry Populations Identifies Putatively Functional Loci. Cancer Epidemiol Biomarkers Prev. 2016;25(12):1609-18.
15. Li N, Johnson DC, Weinhold N, Kimber S, Dobbins SE, Mitchell JS, et al. Genetic Predisposition to Multiple Myeloma at 5q15 Is Mediated by an ELL2 Enhancer Polymorphism. Cell Rep. 2017;20(11):2556-64.
16. Du Z, Weinhold N, Song GC, Rand KA, Van Den Berg DJ, Hwang AE et al. A meta-analysis of genome-wide association studies of multiple myeloma among men and women of African ancestry. Blood Advances. 2020;4(1):181-90.
17. Genomes Project C, Auton A, Brooks LD, Durbin RM, Garrison EP, Kang HM, et al. A global reference for human genetic variation. Nature. 2015;526(7571):68-74.
18. Howie BN, Donnelly P, Marchini J. A flexible and accurate genotype imputation method for the next generation of genome-wide association studies. PLoS Genet. 2009;5(6):e1000529.
19. Li H. Aligning sequence reads, clone sequences and assembly contigs with BWA-MEM. arXiv:13033997. 2013.
20. McKenna A, Hanna M, Banks E, Sivachenko A, Cibulskis K, Kernytsky A, et al. The Genome Analysis Toolkit: a MapReduce framework for analyzing next-generation DNA sequencing data. Genome Res. 2010;20(9):1297-303.
